# Mania a Potu

**Published:** 1877-07

**Authors:** H. L. Wilson

**Affiliations:** Atlanta, Ga.


### MANIA A POTU.

By H. L. WILSON, M.D.

(*Read before the Atlanta Academy of Medicine.*)

This is a subject by no means new to the present generation. Indeed we are told that as far back as the memory of man runneth, vinous potations have been indulged in to excess. Now, is the moderate use of the pure grape juice hurtful to mankind—when not contraindicted by disease? It seems there is nothing more grateful to the inner man, when worn down by fatigue and physical exhaustion, than a "pony" glass of wine. Some are even sure to smile and moisten their lips, as the eye sparkles upon the introduction of the capacious and flowing "schooner." It is not the prudent or judicious use of alcoholic fermentations that produces mania or delirium, but the continuous introduction into the stomach of vinous or alcoholic drinks, during days or weeks, thereby causing excessive stimulation of the cerebrum, which in turn causes cardiac excitement, creating an abnormal condition of the brain, as well as the entire circulation.

As the drinking continues, there is a disposition to congestion of the liver, with a general perturbation of all the secretions of the alimentary canal, while the kidneys are placed as it were, upon double duty, by this cardiac stimulation. We are usually sent for to see a patient when he becomes sick to nausea, tremulous, and sometimes dangerous, and the stomach positively refuses to receive any more of this "fire-water." In most cases we find an exceedingly penitent individual, constantly taking fluids, and as promptly rejecting them; the entire nervous system highly excited, giving one the appearance of general chorea; pulse from 120 to 130 or 140, causing a harsh, dry skin, parched tongue, with an intolerable thirst. Just here we are informed that the sufferer intends immediate reformation. He denounces whisky and all kindred drinks as a vile fraud, and declares his intention of becoming a blatant advocate of temperance for all future time, reminding you of the kind and confiding female, who so solemnly announces to her friends, during the act of parturition, that her first child will certainly be her last. And strange as it may seem, these positive and vehement declarations, are received with but a moiety of faith, by the older hearers, for the vow of the one is about as soon broken, as that of the other.

At this period I would prescribe, mild chloride of mercury, grs. xxx, with opium, gr. i, unless the liver had been long inactive from debauch, when we might with propriety double the dose of mercury. On seeing the patient again we are informed by him—probably in a whisper—that his chamber is infested with reptiles and animals, who, with their impudent and bleary eyes, are peering into his inmost soul. Sometimes he sees men with short legs, big feet, long bony arms, and immense eye balls, who solemnly march to and fro watching his every movement. Sometimes the patient becomes so desperate in his mental perturbations that he would commit violence under the belief that he was protecting his own life from assassination. Hence, caution is necessary on entering their apartments when they are alone.

Of course sleep is out of the question. In some instances opium and small doses of whisky will give them quiet and rest; but in my opinion it is best to abandon the alcoholic stimulant, and as soon as you get the desired action of the calomel, reduce the pulse with Norwood's veratrum: say three drops every two or three hours. However, we must administer with care or we may reduce too rapidly. As soon as we have it down to 75 or 80 beats per minute, I would give bromide of calcium, grs. xx, and at the expiration of one hour if no sleep, give chloral hydrate, grs. xv. Then if sleep be not induced in two or three hours double the dose of each, giving the latter *one* hour after the former as before. Good results often follow the use of mustard applied to the epigastrium, in allaying the irritability of the stomach. By the above treatment I think we may confidently expect the liver to be freely emulged, sleep and quiet produced, with re-established secretions, moist skin, normal mental condition, etc. I would then advise soups, iced milk, broth or toast-water until the stomach could retain more substantial food. I believe it is the *sudden* withdrawal of the cerebral stimulant which the brain for the time had been educated to receive, that produces the mental hallucination and phantoms that appear and vanish.

